# "Carriers" of Disease

**Published:** 1921-05-21

**Authors:** 


### "Carriers" of Disease.

South Africa is making it compulsory for "carriers" of disease to be notified, in order that due surveillance may be exercised by the local authorities. The Minister of Public Health is now asking municipal authorities to submit for promulgation regulations in respect of "carriers" of notifiable infectious disease to be framed under the 1919 Public Health Act.

The draft regulations mark an epoch in health administration, and therefore warrant publicity in detail. "Carriers" are defined as persons who, though not at the time suffering from a notifiable infectious disease, are, as proved by laboratory examination and investigation, harbouring the infection of, and consequently liable to cause the spread of, such disease.

Any person believed or suspected on reasonable grounds by the medical officer of health of the local authority, or by a health officer or medical officer of the Government, to be a "carrier" shall afford to such officer every facility for obtaining specimens of blood, excreta, discharges, or other material required for examination and investigation, and shall take any medicine prescribed by such officer for that purpose.

Where it is certified that any person is believed to be a "carrier," and that necessary examination cannot be properly carried out at such person's home, the magistrate may make an order requiring such person to proceed or be removed to a hospital or other suitable place for the purpose of examination and investigation, and to remain or be detained therein for such reasonable period as may be required for that purpose.

Every "carrier" shall at all times observe and give effect to all reasonable practicable instructions in regard to the disposal of his excreta, the cleansing of articles used by him, or other precautions for preventing the spread of infection.

Such "carrier" shall inform the local authority of his intention to change his place of residence or work and of his intended new place of residence or work.

Where, on the certificate of the medical officer, it appears to the magistrate that a person is a "carrier," the magistrate, on the application of such officer and after due inquiry, may, having regard to the nature of the infection and any material assistance which the local authority or the Government is prepared to give to mitigate hardship to the individual or his dependants, make, and may from time to time modify, alter, extend, or rescind an order or orders requiring such person (*a*) to proceed or be removed to, and to remain or be detained for a period to be specified in, a hospital for the purpose of medical treatment; (*b*) to attend regularly for treatment or examination at times and places specified; (*c*) to proceed to and remain in a specified locality under medical surveillance for a period specified, and, if considered necessary, to attend and report himself at specified times and places; (*d*) not to handle or otherwise come in contact with food or vessels or articles containing or used to contain food intended for consumption by others, or to engage in any occupation entailing the handling or coming in contact with such food, vessels, or articles; (*e*) to comply with such other requirements as are deemed necessary for safeguarding the public health.

Medical officers and magistrates must ensure that regulations are carried out sympathetically and without more hardship to any person than is necessary and unavoidable in the public interest.

Any person found guilty of a contravention of or failure to comply with the regulations, or failure to assist in their enforcement, will be liable to penalties.

